# Relationship of urinary endothelin-1 with estimated glomerular filtration rate in autosomal dominant polycystic kidney disease: a pilot cross-sectional analysis

**DOI:** 10.1186/s12882-016-0232-8

**Published:** 2016-02-29

**Authors:** Rupesh Raina, Linda Lou, Bruce Berger, Beth Vogt, Angelique Sao-Mai Do, Robert Cunningham, Pauravi Vasavada, Karin Herrmann, Katherine Dell, Michael Simonson

**Affiliations:** Department of Pediatrics, Division of Pediatric Nephrology, Rainbow Babies and Children’s Hospital, Cleveland, USA; Department of Medicine, Division of Nephrology and Hypertension, Cleveland, USA; Department of Radiology, Cleveland, USA; University Hospitals Case Medical Center, Center for Pediatric Nephrology, Cleveland Clinic Foundation, Cleveland, USA; Case Western Reserve University School of Medicine, 2109 Adelbert Road, Biomedical Research Bldg. #322, Cleveland, OH 44106 USA

**Keywords:** Autosomal dominant polycystic kidney disease, Endothelin-1, Cyst growth, Hypertension, Kidney volume

## Abstract

**Background:**

The pathogenesis of progressive renal insufficiency in autosomal dominant polycystic kidney disease (ADPKD) is unclear. Evidence from experimental models of ADPKD suggests that elevated endothelin-1 (ET-1) drives cyst growth, renal fibrosis and loss of renal function, but whether ET-1 is elevated in humans with ADPKD is uncertain.

**Methods:**

In a cross-sectional study of ADPKD we measured urinary ET-1, a surrogate for ET-1 in kidney cortex, in spot collections corrected for creatinine. The volume of each kidney was measured using MRI-based stereology. The relationship of urine ET-1 with MDRD eGFR and kidney volume was modeled by multiple linear regression with adjustment for clinical covariates.

**Results:**

Patients with ADPKD were ages 18 to 53 with eGFRs (median, interquartile range) of 63.2 (43.5–80.2) ml/min/1.73 m^2^ and albumin/creatinine ratios (ACR) of 115.0 (7.5–58.5) μg/mg. Urine ET-1 was inversely associated with eGFR (*r* = −0.480, *P* < 0.05) and positively (*r* = 0.407, *P =* 0.066) with ACR independent of age and female sex (*P* < 0.01). ET-1 appeared to be positively associated with total kidney volume (*r* = 0.426, *P* = 0.100), with a test for trend across urine ET-1 quartiles yielding *z* = 1.83, *P* = 0.068. ET-1 strongly correlated with NAGase (*r* = 0. 687, *P* = 0.001), a marker of tubular damage and a surrogate marker of renal disease progression in ADPKD. Of note, ET-1 levels in urine were not correlated with hypertension.

**Conclusions:**

In a translational study of patients with ADPKD, urinary ET-1 was inversely associated with eGFR and positively correlated with total kidney volume. Taken together with results from experimental models, these findings suggest that the role of ET-1 in ADPKD warrants further investigation.

**Electronic supplementary material:**

The online version of this article (doi:10.1186/s12882-016-0232-8) contains supplementary material, which is available to authorized users.

## Background

Autosomal dominant polycystic kidney disease (ADPKD) is the most prevalent inherited form of kidney disease and the fourth leading cause of end-stage renal disease (ESRD) in the US [1, 2]. ADPKD occurs in one of every 400 live births and is characterized by bilateral, progressive enlargement of focal cysts in all nephron segments. Onset and morbidity often occurs in children and adolescents and leads to numerous complications including hypertension, pain, hematuria, proteinuria, kidney stones and declining renal function. Direct medical costs for dialysis and transplantation to treat ADPKD are estimated at 1.5 billion U.S. dollars per year [2]. Current therapies are directed at controlling hypertension, pain and the complications of chronic kidney disease but do not prevent progression of ADPKD [3].

Endothelin-1 (ET-1), an endothelium-derived vasoconstrictor and pro-fibrotic peptide [4–6], has emerged as a possible therapeutic target for inhibiting cyst growth and interstitial fibrosis in ADPKD. Transgenic overexpression of ET-1 in mice causes extensive cyst formation in the kidney with glomerular and peritubular fibrosis and monocyte infiltration without increasing blood pressure [7]. Renal expression of ET-1 is robustly elevated in murine and rat models of PKD and is associated with cyst formation and the development of hypertension [8–10]. In humans with ADPKD, immunoreactive ET-1 has been localized in cyst epithelia, mesangial cells and vascular smooth muscle cells [11]. Compared to healthy age-matched controls, ET_A_ mRNA is 5-10-fold higher in ADPKD cystic kidneys. In a small cohort of patients with all forms of PKD (*n* = 20, ESCAPE Trial Group), urine ET-1 excretion was elevated compared to control subjects without chronic kidney disease [12].

Synthesis of ET-1 by kidney cells is the primary source of urinary ET-1; < 1.0 % of ET-1 in urine derives from the filtered load [13–17]. Thus urinary ET- is a non-invasive surrogate for ET-1 in kidney tissue that avoids the risk and cost associated with immunohistochemical analysis in percutaneous renal biopsy. Here, we investigated the hypothesis that elevated levels of urine ET-1 are associated with decreased eGFR and increased total kidney volume in a pilot study of outpatients with ADPKD.

## Subjects and methods

### Study protocol

This was a cross-sectional study of 21 ADPKD outpatients recruited between April 2013 and February 2014 at University Hospitals Case Medical Center (Additional file 1). Studies were approved by the Institutional Review Board at University Hospitals Case Medical Center, and informed consent was obtained from all participants. Eligibility criteria included diagnosis of ADPKD by the Modified Ravine Unified Criteria [18] and eGFR ≥ 15 ml/min/1.73 m^2^ estimated by the MDRD formula [19]. Patients were diagnosed with ADPKD if radiographic imaging demonstrated at least two renal cysts in the setting of a family history of ADPKD, or in families of unknown genotype, the presence of three or more renal cysts (in individuals between 15 and 39 years old), or two or more cysts in each kidney (in individuals between 40 and 59 years old). Exclusion criteria included unwillingness or inability to provide informed consent, pregnancy, lactation, and current substance abuse. We also recruited seven age- and sex-matched controls without apparent kidney disease. All data were obtained at the study visit or from the paper or electronic medical record. Blood pressure was averaged from three consecutive measurements (1 min between readings), with the patient seated using an automated oscillometric device (Omron HEM-907). Hypertension was defined as systolic BP ≥ 140 mmHg, diastolic BP ≥ 90 mmHg, or use of antihypertensive medications (thiazide diuretics, β-blockers, calcium channel blockers, angiotensin-converting enzyme inhibitors, angiotensin type 2 receptor blockers). Polycystic liver disease in patients with ADPKD was defined as the presence of ≥ 4 cysts on MRI or ultrasound [20].

### Urine collection and processing

Trained personnel obtained random spot urine specimens (∼40 ml midstream) at study visits. Aliquots of urine were sent to the University Hospitals Case Medical Center central laboratory for measurement of urine albumin and creatinine. Remaining urine (~30 ml) was processed as described by the Human Kidney and Urine Proteome Project and the European Urine and Kidney Proteomics Initiative [21]. After conventional urine dipstick analysis (Multistix 8 SG, Bayer, Tarrytown, NY), the specimen was transferred to a 50-ml conical tube and centrifuged at 1500 × *g* for 10 min in a refrigerated centrifuge to pellet cells. Smaller aliquots (10 ml) were centrifuged at 10,000 × *g* to remove particulates and aliquoted for storage at −80 °C. Processed samples were frozen within 3 h of collection. Before analysis, urine pH was adjusted to 8.0 with 1 M Tris buffer (pH 8.0) to help solubilize aggregates that may form after thawing. Assays were performed within 4 months of urine collection after no more than 1 freeze-thaw cycle.

### Measurements of total kidney volume

Magnetic resonance imaging studies were performed as previously reported by Chapman et al. in the CRISP study of ADPKD [22]. Briefly, we obtained coronal T2-weighted images and gadolinium-enhanced three-dimensional volume-interpolated spoiled-gradient echo coronal T1-weighted images (3-mm slice thickness). Kidney volumes were measured in T1-weighted images by stereology estimated from contiguous images by summing the products of the area measurements and slice thickness. Reliability coefficients were 0.972 for TKV in repeatedly acquired images on individual patients. The average coefficients of variation of TKV is estimated to be 0.01 [23].

### Sandwich enzyme-linked immunosorbent assay of human ET-1

Urinary ET-1 was measured in duplicate by ELISA (R&D Systems, Minneapolis, MN) as previously reported [17]. Mean ET-1 concentration was normalized for urine creatinine measured by the University Hospitals Case Medical Center central laboratory. Urine aliquots for measurement of ET-1 were extracted in acetic acid exactly as described [24]. The intra- and interassay coefficient of variation was equal to or less than the manufacturer-reported values in human urine. Laboratory personnel were blinded. Biological variation in urine ET-1 excretion in ADPKD participants was calculated in ten randomly selected participants with three serial measurements over 24 weeks as the intra-(CV_I_) and inter-individual (CV_G_) coefficient of variation as described by Fraser [25].

### Urinary excretion of NAGase

N-acetyl-β-D-glucosaminidase (NAGase) is a 130-kD glycolytic lysosomal enzyme localized mainly in proximal tubules and shed into urine upon cell injury in a variety of kidney diseases [26, 27]. We measured urine NAGase using the substrate 2-methoxy-4-(2-nitrovinyl) phenyl-glucosaminide and bovine NAGase as a calibrant according to the manufacturers’ protocols (PPR Diagnostics, London). NAGase values were corrected for urine creatinine. The intraassay and interassay coefficients of variation were 8.3 and 12.3 %, respectively.

### Statistical analysis

Continuous variables are presented as median and interquartile range (IQR) and proportions for categorical variables. Normality was assessed by visual inspection and the Shapiro-Wilk test, *P* > 0.05. The distribution of urine ET-1 was highly skewed leftward, so analyses were performed on log-transformed data. Pearson correlation of urine ET-1 with MDRD eGFR and total kidney volume are presented. Median total kidney volume was compared across quartiles of urine ET-1 using the test for trend across ordered groups described by Cuzick [28]. The association of urine ET-1 with eGFR and ACR was assessed in multiple variable linear regression models that included age and sex as potential confounders. Data analysis was performed by the authors (LL, AS-D, and MSS) using Stata 13.0 (Stata, College Station Texas, United States).

## Results

Twenty-one participants were recruited with median age of 39.2 years; 29 % were female (Table 1). Median eGFR was 63.2 ml/min/1.73 m^2^ and albumin to creatinine ratio was 115 μg/mg. Values for systolic blood pressure were under 140 mm Hg with a median of 123 mm Hg (Table 1). Twenty-two percent of participants had polycystic liver disease, and 82 % were taking renin angiotensin system inhibitors or Tolvaptan (Table 1).

**Table 1:** Clinical characteristics of the cross-sectional sample of patients with ADPKD, *n* = 21

Characteristic	Median (IQR^a^)
Age	39.2 (25–50)
Female (%)	29
eGFR, ml/min/1.73 m2	63.2 (43.5–80.2)
Urine Albumin/Creatinine Ratio, μg/mg	115.0 (7.5–58.5)
Total Kidney Volume, ml	1270 (839–1545)
Systolic Blood Pressure, mm Hg	123 (110–138)
Diastolic Blood Pressure, mm Hg	88 (79–98)
Polycystic Liver Disease (%)	22
Renin Angiotensin System Inhibitors (%)	82
Tolvaptan (%)	82

^a^ *IQR* interquartile range

### Correlation of urine ET-1 with eGFR

**Fig. 1:**
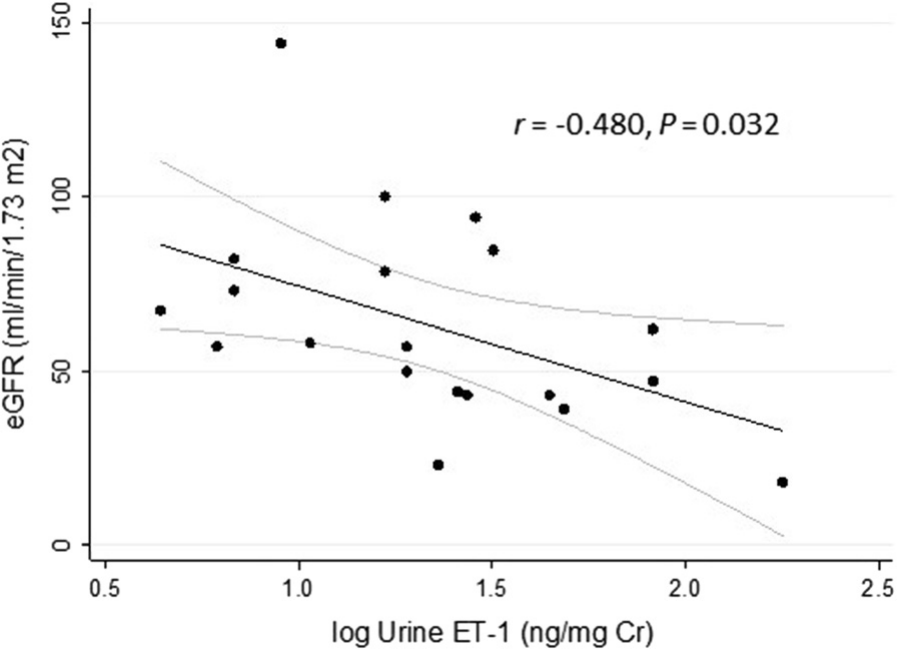
Urine ET-1 inversely correlated with eGFR in a cross-sectional analysis of 21 patients with ADPKD. ET-1 was measured by ELISA in spot urine specimens and values were adjusted for urine creatinine. Because the distribution of urine ET-1 was non-normal, log-transformed values were utilized

Urine ET-1 was elevated in participants with ADPKD compared to age- and sex-matched controls with without apparent kidney disease (mean ± SD, 4.1 ± 2.9 versus 1.3 ± 0.9 ng/mg creatinine, *P* < 0.01). In participants with ADPKD, urine ET-1 was associated with lower eGFR. A Pearson’s product–moment correlation was run to assess the relationship between urine ET-1 and eGFR. Preliminary analysis showed the relationship to be linear with log transformed urine ET-1 normally distributed as assessed by the Shapiro-Wilk test (*P* < 0.05). As shown in Fig. 1, there was a moderate inverse correlation, *r* = −0.480, *P* = 0.026, with urine ET-1 explaining 25 % of the variation in eGFR. The biological variation of urine ET-1 excretion in ADPKD participants was assessed in ten randomly selected participants with three serial measurements over 24 weeks. The intra- individual coefficient of variation (CV_I_) was 12.8 % and inter-individual coefficient of variation (CV_G_) was 14.6 %, making it unlikely that natural variation in ET-1 excretion does not account for the magnitude of the inverse association of urine ET-1 with eGFR [25]. ET-1 correlated with log albuminuria, *r* = 0.407, but did not quite reach statistical significance at *P* = 0.066. Interestingly, urine ET-1 did not correlate with systolic or diastolic blood pressure.

A multiple linear regression was run to test for possible confounding between urine ET-1 and eGFR or ACR by age and sex. The assumptions of linearity, independence of errors and normality of residuals were met. The variables statistically significantly predicted eGFR, F(3,16) = 4.90, *P* = 0.013, adjusted *R*^*2*^ = 0.38. Only urine ET-1 and age added statistically significantly to prediction of eGFR, *P* < 0.05. However, the contribution of age was modest, as demonstrated by the regression coefficients and standard errors (Table 2). Holding urine ET-1 and sex constant, eGFR is predicted to decrease by 1.0 ml/min/1.73 m^2^ when age increases by 1 year, whereas holding age and sex constant, eGFR is predicted to decrease 5.4 ml/min/1.73 m^2^ for every 1 ng/mg increment in urine ET-1. In separate regression models the regression of eGFR on urine ET-1 was not statistically significantly affected by treatment with renin angiotensin system inhibitors or Tolvaptan (data not shown). Similarly, there was a small but statistically significant effect of age on prediction of ACR (Table 2), and no statistically significant effect of renin angiotensin system inhibitors or Tolvaptan.

**Table 2:** Summary of multiple linear regression model of ET-1, age and sex to predict eGFR and ACR in patients with ADPKD

		eGFR			ACR	
Variable	*B*	SE_*B*_	*Π*	*B*	SE_*B*_	*P*
Urine ET-1	−5.366	2.910	0.084	104.9	36.1	0.023
Age	−1.017	0.388	0.019	−14.7	6.1	0.041
Sex	8.978	12.541	0.484	−118.2	152.7	0.461

Note: * *P* < 0.05; *B* unstandardized regression coefficient, *SE*_*B*_ standard error of *B*. Overall *P* for both models is *P* < 0.01

### Urine NAGase and ET-1 levels

Damage to the structure and function of tubular epithelial cells characterizes ADPKD and often precedes the decline in GFR [1]. Therefore, we examined the correlation urine NAGase, an index of tubular damage [27], with ET-1 excretion. ET-1 strongly correlated with NAGase (*r* = 0. 687, *P* = 0.001). Urine NAGase was moderately and inversely associated with eGFR (*r* = −0.551, *P* = 0.001) and strongly with total kidney volume (*r* = 0.728, *P* = 0.001).

### Urine ET-1 and total kidney volume

**Fig. 2:**
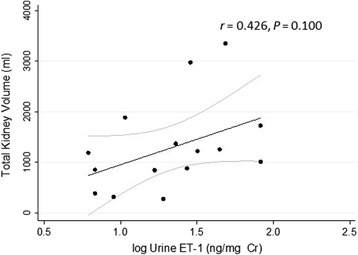
Positive correlation of urine ET-1 with total kidney volume. The log transform of urine ET-1 measured in spot urine specimens was plotted versus total kidney volume, *r* = 0.426, that did not attain statistical significance, *P* = 0.100. Identical results were obtained when TKV was referenced to participant height

**Fig. 3:**
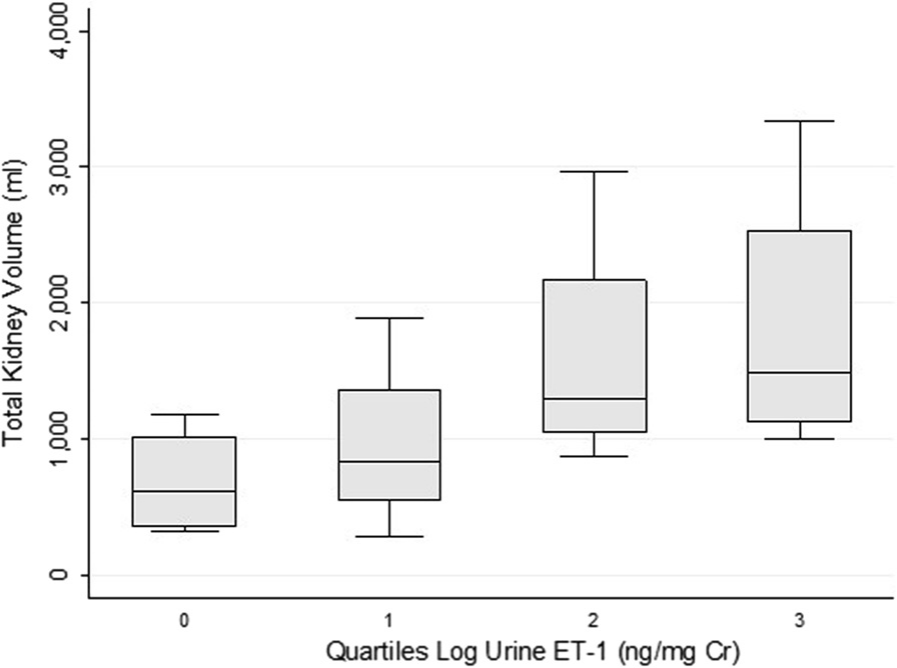
Total kidney volume trended higher across quartiles of urine ET-1. Each box represents the 25^th^ to 75^th^ percentile, and the line represents the median total kidney volume within a given quartile of log urine ET-1. There was a trend towards higher total kidney volume as urine ET-1 increased, *z* = 1.83, *P* = 0.068

Total kidney volume at baseline predicts risk of developing renal insufficiency [23], and in our study there was a trend towards lower eGFR in patients with higher total kidney volume, *r* = −0.289, *P* = 0.278. Because ET-1 stimulates cyst formation and growth in animal models, we examined the relationship of urine ET-1 with total kidney volume, an index of cyst growth. Higher levels of urine ET-1 were correlated with increased total kidney volume in patients with ADPKD (Fig. 2). There was a moderate positive correlation of urine ET-1 with total kidney volume, *r* = 0.426, that did not attain statistical significance, *P* = 0.100, with ET-1 explaining 16 % of the variation in total kidney volume. To further evaluate the relationship between urine ET-1 and total kidney volume, we tested for trend (Fig. 3). Testing for trend using a nonparametric test across quartiles yielded *z* = 1.83, *P* = 0.068.

## Discussion

Abundant preclinical data suggest that elevated ET-1 promotes cyst growth and renal fibrosis in ADPKD [7–10]. Here we report that increased ET-1 was associated with decreased eGFR and may be correlated with elevated TKV in patients with ADPKD. ET-1 correlated with a marker of tubular damage in ADPKD, NAGase, but not with systolic or diastolic blood pressure. Taken together with data from experimental models, these results support the hypothesis that ET-1 is elevated in patients with ADPKD is association with renal insufficiency.

Grenda et al. [12] reported a 1.6-fold increase in urinary ET-1 excretion compared to controls in 20 children (age 10.2 **±** 3.6 years) with polycystic kidney disease. In the present study, we significantly expand on these results by showing that urine ET-1 is elevated 3.2-fold in adults with ADPKD compared to age- and gender-matched controls. Urinary excretion of ET-1 in ADPKD was significantly and negatively associated with eGFR (*r* = −0.480, *P* = 0.026), and appeared to be positively associated with increased excretion of urine albumin, consistent with a role in progressive renal injury and dysfunction. Of note, urine ET-1 did not correlate with hypertension, which further supports results in experimental models demonstrating a blood pressure-independent action of ET-1 in the pathogenesis of ADPKD [7, 10].

Elevated ET-1 has correlated with scarring and chronic disease in a variety of preclinical models of kidney disease [10, 11, 29, 30], thus we asked whether ET-1 correlates with urine NAGase in ADPKD. ET-1 correlated strongly with NAGase (*r* = 0. 687, *P* = 0.001). These findings may be significant because ADPKD is characterized histologically by injury to tubules that often precedes a decline in GFR [1]. NAGase, distributed mainly in lysozymes of the proximal tubule and cyst-lining epithelial cells in ADPKD patients [26, 31], is shed into urine upon damage to tubular epithelial cells [27]. In a cross-sectional analysis, urine NAGase was 7.8-fold higher in 102 participants with ADPKD compared to 102 age- and sex-matched healthy controls [32]. Moreover, urine NAGase predicted patients with progressive decline of GFR (area under the receiver operating characteristic curve = 0.794) in a 1-year prospective cohort study of 270 patients with ADPKD [31]. Consistent with previous studies by Meijer et al. [32] and Park et al. [31], we also observed that NAGase correlated inversely with eGFR and positively with total kidney volume. Collectively, these data are consistent with a role for ET-1 as a mediator of tubular damage in ADPKD.

Progression of ADPKD stems primarily from formation and growth of renal cysts [33, 34]. Cyst formation, due to localized epithelial cell proliferation, results in renal function decline secondary to obstruction of the tubules in which cysts grow. Cyst growth is also thought to be responsible for subsequent upstream blockage, downstream tubular atrophy and, eventually, glomerulosclerosis. Thus, while cyst formation is an early indicator of disease progression, in the later stages, fibrosis is the dominant factor for renal decline [34]. Current therapies are directed at controlling hypertension, pain and the complications of chronic kidney disease but do not prevent progression of ADPKD [3]. In addition, standard measures of renal disease progression, including estimated or measured glomerular filtration rates (GFR), do not show changes until significant damage has occurred. Thus, there is an urgent need for non-invasive biomarkers to identify ADPKD patients at high risk for progression before significant renal insufficiency occurs. Those patients, at an early stage of disease, could then be targeted for therapies as new approaches emerge. It is also possible that ET-1 may be a therapeutic target in ADPKD. In a previous study, an ET_B_ receptor antagonist (A-192621) accelerated disease progression in a murine model of ADPKD [35]. However, recent studies demonstrate differential effects of ET_A_ and ET_B_ receptors in progression of kidney disease and favor a reno-protective effect of ET_A_ receptor antagonists [36], so studies are necessary to determine efficacy of ET-1 receptor blockers in ADPKD.

## Conclusions

Urine ET-1 is associated inversely with eGFR independent of age, sex and blood pressure in a pilot, cross-sectional study. Potential significance of our findings are that ET-1 may be a novel therapeutic target for slowing progression of kidney disease in ADPKD. Development of orally-active receptor antagonists for ET_A_ and/or ET_B_ receptor is in early stages, but these drugs have been approved recently for treatment of pulmonary artery hypertension and scleroderma-related digital ulcers. Multiple clinical studies point to efficacy of ET_A_ receptor antagonists in diabetic and non-diabetic chronic kidney disease [37, 38]. To our knowledge ET_A_ or ET_B_ antagonists have not been studied in trials of ADPKD. Another possibility is that elevated urinary excretion of ET-1 may be a biomarker of early renal injury. Our findings suggest that additional studies of ET-1 and ET-1 receptor antagonists in ADPKD, particularly longitudinal trials, may be warranted.

## Additional file

Additional file 1:
**STROBE Statement—Checklist of items that should be included in reports of** ***cross*** **-** ***sectional studies.*** (DOC 79 kb)

## Abbreviations

ACR: albumin/creatinine ratio
ADPKD: autosomal dominant polycystic kidney disease
eGFR: estimated glomerular filtration rate
ET-1: endothelin-1
NAGase: N-acetyl-β-D-glucosaminidase
TKV: total kidney volume
